# The League of Mercy

**Published:** 1900-12-08

**Authors:** 


### THE LEAGUE OF MERCY.

The hon. secretaries and the treasurer of the League make the following appeal:—

The recent appeal of the Prince of Wales's Hospital Fund to the public for subscriptions raises the question of the wants of the London hospitals. This fund has been productive of enormous benefit to the hospitals, not only in the direction of financial assistance, but by causing improvements and reforms to be carried into effect.

The Prince of Wales's Hospital Fund has mainly been supported by generous donors and subscribers of large amounts.

It is known that there are vast numbers of persons who, willing and in many cases anxious to contribute, are unable to give largely; and to meet these cases the Prince of Wales established the League of Mercy—now founded by a Royal Charter. The constitution of the League is so arranged that contributions of one shilling or upwards may be made through members of the League, who, working under presidents and vice-presidents in all parts of London and the home counties, are in a position to obtain help for the hospitals which would not otherwise be received.

If everyone who does not in any other way subscribe would contribute one shilling to the League of Mercy through a member, the needs of the hospitals would be supplied. In this way the special appeals from hospitals which from time to time are made would not be required. It would be a distinct advantage if these special appeals could be avoided. They have many objectionable features—one being that they dislocate the sources of charity, as the War and Famine Funds have done during the past year.

It may be stated that every farthing of every shilling given to the League goes direct, without any deduction for expenses, for the benefit of the London hospitals.

The League of Mercy was organised to secure some measure of personal service on the part of a large number of persons who are sufficiently conscious of the value of our London hospitals, and are willing to devote a little time and trouble to bring their needs and the services which they render to the public to the notice of those who have hitherto, from want of opportunity or knowledge, taken no interest in this work. The organisation of the League has now permeated throughout London and the Home Counties, and we would be very glad to have offers of personal service or to give information as to how good work can be done by anyone who is prepared to give a little time each year on behalf of the hospitals.

